# Working Environment and Morbidities of Child Laborers in an Urban Slum of Kolkata

**DOI:** 10.4103/0970-0218.43242

**Published:** 2008-10

**Authors:** Indira Dey

**Affiliations:** Department of Community Medicine, North Bengal Medical College, Darjeeling, India. E-mail: indiradeypal@rediffmail.com

Sir,

According to the World Health Report (1995), 15% of children aged 10–14 years old were working in Asia and India has the largest percentage of child laborers in the world.(1) Child labor contributes to about 20% of India's GNP(1) and mostly operates in the unorganized, informal, and unregulated sectors of the economy and is not being adequately reported. The most powerful force driving children into labor is exploitation of poverty. They begin to work at a very young age. These child laborers are engaged in various types of working situations in unorganized urban sectors. Poor and unsafe working conditions adversely affect these children and they may suffer from poor physical, mental, and social development. They have to work in an unkind, uncomfortable, and often physically hazardous environment for long hours. This study was carried out to assess the working conditions and environment of child laborers and to determine their morbidity pattern.

A cross-sectional, observational study was conducted for a period of 3 months from October to December 2003 in the service area of the Urban Health Centre (UHC), Chetla, Kolkata under the All India Institute of Hygiene and Public Health, Kolkata. Of the four sectors catered by this UHC, Sector I and III were selected randomly for the study. Children working in two lanes of each sector, chosen randomly, were considered for the study. The study units were selected based on the definition given by the Operation Research Group, Baroda i.e., children between 5–14 years old, full-time workers, and on remunerative work.(2) The children working in commercial establishments were considered for the study. Based on this, the sample size came to be 45.

Data was collected using a pretested and predesigned schedule. The children were interviewed in their working places. The adult workers were interviewed to judge the validity of information. Observation was done to assess the working place environment such as, the type of construction, ventilation, lighting, availability of drinking water, latrine facility, etc. The current morbidity pattern was recorded based on 2 weeks recall.

The study revealed that a majority (86.6%) of the child laborers were male and most of the children (84.4%) were between the ages of 11 and 14 years old. The average age of the child laborers was 11.9 years old. Among the working children, 73.3% were Hindus and the rest were Muslims. A majority (84.4%) of the children were from nuclear families. For 6.7% of the children, both the parents were dead and in the case of 17.7% of the children, the father was either dead or had left the family. A total of 48.9% of the children had not been to school, 46.7% of the children received primary education, and only 4.4% of the children had gone to middle school. A total of 5 out of 45 children were still trying to pursue their education.

Among the study population, 42.3% worked in a garage, 35.5% were rag pickers, 13.3% worked in hotels and food stalls, and 8.9% were shop helpers. None of the children were engaged in so-called hazardous occupations as described by the Indian Child Labor (Prohibition and Regulation) Act, 1986. Children working in garages, hotels, and shops were all males and the females were rag pickers.

The Indian Child Labor (Prohibition and Regulation) Act, 1986 had recommended that the child workers would work for a maximum of 6 hours a day with 1 hour rest after 3 hours of work and they would get a weekly holiday. The present study showed [Table 1] that most of the child laborers (71.2%) worked for more than 6 hours a day and about one third of them did not get any rest in between their work. All the child laborers included in the study get one weekly holiday except the hotel boys who are allowed leave after one or two months of continuous work or when they are sick.

**Table 1:** Distribution of child laborers according to working hours and remuneration

	Garage workers	Rag pickers	Hotel boys	Shop helpers	Total
Working hours					
4 to 6	—	13 (28.8)	—	—	13 (28.8)
7 to 9	16 (35.6)	3 (6.7)	—	3 (6.7)	22 (49.0)
10 to 12	3 (6.7)	—	6 (13.3)	1 (2.2)	10 (22.2)
Remuneration/month (Rs)					
>=300	—	4 (8.8)	5 (11.1)	3 (6.7)	12 (26.6)
301-500	8 (17.8)	9 (20)	1 (2.2)	1 (2.2)	19 (42.2)
>500	11 (24.5)	3 (6.7)	—	—	14 (31.2)

Figures in parentheses are in percentages

It was found that 26.6% of the child laborers were paid less than Rs 300 per month. Among the laborers working, 21 (46.7%) received food, mainly hotel and some garage workers and 6 (13.3%) received tips. Financial support in medical care was available to 20 (44.4%) of the workers. The majority of the working children had to spend almost all of their income to support their family. A total of 32 (71%) of the working children were satisfied with their working conditions, 17.8% felt tired during work, and 11.1% considered the remuneration to be very low.

As laid down in the Child Labour Act, it is the responsibility of the appropriate government agencies to make rules to provide safe and healthy environmental conditions for child laborers such as disposal of wastes and effluents, ventilation, temperature, lighting, cleanliness, drinking water, latrine, urinals, fencing of machinery, etc. Among the study population, 35.5% of the children worked totally exposed to the sun and rain and 31% had to work partly under open air. Almost all the working places were adequately ventilated and illuminated. A total of 44.4% had no latrine facilities at their work places although arrangements for drinking water were present in most (84%) of the places.

Being fragile physically, children are more susceptible than adults to various work-related injuries and illnesses. Also, because they are not yet matured mentally, they are less aware, even completely unaware, of the potential risks involved in their specific occupations or at the work place itself. About 40% of the children said that they had no health problems in last 2 weeks but 31% complained of respiratory infections, 24% had skin infections, and 13.3% had toothaches and caries tooth. A total of 42.2% had pallor and 11.1% had signs of vitamin B deficiency. Physical injury while working had been sustained by 6 (13.3%) children. Morbidities were more in children who worked in the sun and rain (87.5%) than those who were not exposed (44.08%) and the difference is statistically significant.

It is now evident that child labor cannot be totally eradicated by legislation alone. Implementation of such laws and detection of violations, especially regarding working hours, rest period, holidays and to supervise the working environment are necessary. We should try to provide these children with education, supplementary nutrition, health care, and vocational training to improve their living conditions.

## References

[1] Park K Text book of preventive and social medicine.

[2] Khatu KK (1983). Working children in India.

